# Antibiotic-Resistant Vibrios in Farmed Shrimp

**DOI:** 10.1155/2015/505914

**Published:** 2015-03-30

**Authors:** Renata Albuquerque Costa, Rayza Lima Araújo, Oscarina Viana Souza, Regine Helena Silva dos Fernandes Vieira

**Affiliations:** ^1^Sea Science Institute, Federal University of Ceará, Avenida Abolição 3207, 60165-081 Fortaleza, CE, Brazil; ^2^Fisheries Engineering Department, Federal University of Ceará, 60440-900 Fortaleza, CE, Brazil

## Abstract

Antimicrobial susceptibility pattern was determined in 100 strains of *Vibrio* isolated from the *Litopenaeus vannamei* shrimp and identified phenotypically. A high antibiotic-resistance index (75%) was observed, with the following phenotypic profiles: monoresistance (*n* = 42), cross-resistance to *β*-lactams (*n* = 20) and multiple resistance (*n* = 13). Plasmid resistance was characterized for penicillin (*n* = 11), penicillin + ampicillin (*n* = 1), penicillin + aztreonam (*n* = 1), and ampicillin (*n* = 1). Resistance to antimicrobial drugs by the other strains (*n* = 86) was possibly mediated by chromosomal genes. The findings of this study support the conclusion that the cultured shrimps can be vehicles of vibrios resistant to *β*-lactam and tetracycline.

## 1. Introduction

Bacteria of the* Vibrio* genus occur naturally in marine, estuarine, and freshwater environments [1] and are notably described as shrimp pathogens [2]. Studies indicate that the antibiotic resistance of vibrios isolated from penaeid culture environment is not unusual [3–5], a fact that apparently constitutes a problem to this type of aquaculture activity [6]. In addition, there is the risk of environmental impact, since the use of antibacterial agents as prophylactic measure in aquaculture favors the selection of resistant bacteria, increasing the probability of transferring resistant genes to human pathogens and land animals [7].

Considering the importance of researching the occurrence of antibiotic-resistant bacteria in marine invertebrates intended for human consumption, this study aimed to determine the susceptibility pattern to antibacterial drugs of vibrios isolated from the hemolymph of* Litopenaeus vannamei* shrimp.

## 2. Materials and Methods

A total of 100* Vibrio* strains from the bacterial collection of the Microbiology Laboratory at the Environmental and Fish-Sea Sciences Institute (LABOMAR-UFC) were used in this experiment. Only strains previously identified by phenotyping were used [8]. The 100 strains, isolated from the hemolymph of* Litopenaeus vannamei*, were subjected to identification by a set of biochemical keys [9], using the following tests: arginine dihydrolase, lysine decarboxylase and ornithine decarboxylase, oxidase, indole,* ortho*-nitrophenyl-*β*-galactoside (ONPG), Voges-Proskauer, D-glucosamine cs, growth at 0%, 3%, 8%, and 10% NaCl, growth at 40° and 4°C, citrate, gelatinase, urease, resistance to O/129, and acid from glucose, sucrose, arabinose, mannitol, and melibiose. The strains had consonant phenotypic profiles with the species* V. navarrensis* (*n* = 53),* V. brasiliensis* (*n* = 15),* V. parahaemolyticus* (*n* = 10),* V. xuii* (*n* = 8),* V. coralliilyticus* (*n* = 5),* V. cholerae* (*n* = 4),* V. neptunius* (*n* = 2),* V. alginolyticus* (*n* = 1),* V. diazotrophicus* (*n* = 1), and* V. vulnificus* B3 (*n* = 1).

All isolates (*n* = 100) were submitted to antibiotic susceptibility pattern tests by disk diffusion method [9]. For the present study, antibiotics used in shrimp industry [4] and human clinical [10] were selected. The following antimicrobials (Laborclin) were tested: nalidixic acid (Nal 30 *μ*g), ampicillin (Amp 10 *μ*g), aztreonam (Atm 30 *μ*g), cephalothin (Cpl 30 *μ*g), ceftriaxone (Cro 30 *μ*g), ciprofloxacin (Cip 5 *μ*g), chloramphenicol (Clo 30 *μ*g), streptomycin (Est 10 *μ*g), gentamicin (Gen 10 *μ*g), imipenem (Ipm 10 *μ*g), nitrofurantoin (Nit 300 *μ*g), penicillin (Pen 10 U) sulfazotrim (Sut 25 *μ*g), and tetracycline (Tcy 30 *μ*g). For the antibiogram test, bacterial density was previously adjusted to a 10^8^ UFC mL^−1^ concentration, by bacterial suspension in saline solution with 1% turbidity equivalent to the McFarland nephelometer scale 0.5. Suspensions with standard densities were inoculated with “swab” in Petri dishes containing Mueller-Hinton Agar (Difco) medium with 1% NaCl, and then antibiotic disks (Laborclin) were applied. The plates were incubated at 35°C/24 h. The inhibition halos were measured (mm) with digital caliper (Digmess) and since there are no breakpoints defined for* Vibrio*, we use the zone diameter interpretive standards for Enterobacteriaceae cited by CLSI [11].

In order to determine the antibiotic resistance mediation, resistant strains were selected and subjected to plasmid curing by acridine orange (Sigma A-6014) [12]. All resistant strains were grown in Luria Bertani broth, supplemented with 0.100 mg mL^−1^ of acridine orange, incubated at 35°C for 24 h. Subsequently, inocula were removed and pour-plated in TSA containing 1% NaCl, incubated at 35°C for 24 h. From TSA growth, antibiogram was performed as mentioned above. The resistance was considered chromosomal when observed after the curing procedure; otherwise, it was characterized as plasmid.

## 3. Results and Discussion

The strains used in this study were subjected to biochemical identification only. For the genus* Vibrio*, the phenotypic identification is still considered not enough, since many species can be misidentified. However, the identification of all strains used was validated [9] and previously published [8].

The resistance to at least one antibiotic was observed in 75 strains (Table 1). The only species susceptible to all drugs was* V. neptunius*. In contrast, resistance was verified and confirmed in all* V. parahaemolyticus, V. xuii*,* V. cholerae*,* V. alginolyticus*,* V. diazotrophicus*, and* V. vulnificus* B3 strains. The isolates of* V. navarrensis*,* V. brasiliensis*, and* V. coralliilyticus* showed high resistance of 71.7%, 60%, and 60%, respectively.

Table 2 describes data related to nine profiles of 75 resistant vibrios. The most frequent profile was the resistance to penicillin alone (*n* = 42), followed by the cross-resistance to *β*-lactams (*n* = 20) and the multidrug resistance (*n* = 13).

Vibrios antibiotic-resistant have been detected in different species of shrimp [4, 6, 13–16]. The expression of this type of resistance is often related to inappropriate use of antibacterial drugs in aquaculture [17, 18].

In a research on antimicrobial susceptibility pattern of* V. parahaemolyticus* derived from shrimp, Bhattacharya et al. [3] revealed the occurrence of strains resistant to ampicillin and sensitive to nalidixic acid and nitrofurantoin. These findings may be compared to the ones from this study, since three strains of* V. parahaemolyticus* were resistant to ampicillin, and all were sensitive to the other antibiotics tested (Table 1). Expression of ampicillin [19] and tetracycline [20] resistance by vibrios from shrimp farming regions has been reported.

Recently, Laganà et al. [21] isolated from Italian aquaculture (fish, shellfish, and crustaceans) sites bacteria (*Vibrio *spp. and* Photobacterium damsela* spp.* piscicida*) resistant to *β*-lactams (ampicillin, carbenicillin, mezlocillin, piperacillin, cephalothin, cefazolin, cefuroxime, cefoxitin, ceftazidime, and aztreonam), quinolones (cinoxacin, nalidixic acid, oxolinic acid, and pipemidic acid), potentiated sulfonamides (sulfamethoxazole + trimethoprim), polymyxin (colistin sulphate), fosfomycin, tetracycline, and RNA synthesis inhibitors (rifampicin). Our results also showed levels of resistance to beta-lactam antibiotics, including cross-resistance (Pen + Cpl, Pen + Atm, Pen + Amp, Cro + Atm, and Pen + Cpl + Amp) (Table 2).

In Brazil,* Vibrio* resistant to antibacterial drugs has been detected in farmed shrimp and cultivation area. Costa et al. [22] detected* Vibrio* strains resistant to ampicillin, sulfazotrim, and ceftriaxone in samples of* Litopenaeus vannamei* shrimp and suggested that the penaeid and its culture environment may constitute the main sources of resistant bacteria. In the present study, a strain with cross-resistance to ceftriaxone and aztreonam was verified; however, there was no resistance to sulfazotrim. Melo et al. [23] reported rates of 90% and 60% antibiotic resistance to ampicillin and amikacin in* V. parahaemolyticus* strains isolated from pond-reared* L. vannamei*. Helena Rebouças et al. [10] showed a high incidence of resistance to ampicillin (45.2%) and to the tetracycline class (38.7%) in vibrios isolated from marine shrimp farming environments.

The high rate (68%) of resistance to penicillin G observed in the present study should be emphasized (Tables 1 and 2). The occurrence of penicillin-resistant vibrios has already been reported in different penaeid culture areas and regions [6, 24]. Srinivasan and Ramasamy [25] detected 100% resistance to penicillin G in vibrios species associated with viral diseased shrimp in India and alert to the environmental, economic, and management problems that may result from the emergence of drug resistant microbial diseases in aquaculture.

Plasmid resistance was characterized for Pen (*n* = 11), Pen + Amp (*n* = 1), Pen + Atm (*n* = 1), and Amp (*n* = 1) (Table 3). Resistance to antimicrobial drugs by the other strains (*n* = 86) was possibly mediated by chromosomal genes.

In the present study only phenotypic detection of plasmids was performed. The curing by acridines has been used since 1960s and normally involves loss of the whole plasmid [26]. In 1970s, Dastidar et al. [27] demonstrated the efficiency of acridine orange in the elimination of R plasmids in* Vibrio cholerae* multidrug-resistant strains. The acridine orange eliminates plasmids from prokaryotic cells [28], and although the conventional methods for curing plasmids by curing agents may induce mutations in the host chromosomal DNA [29], its use in plasmid detection is still being reported in clinical strains [30] and animal/environmental isolates [11, 31, 32].

In 13 strains, the resistance to penicillin G was characterized as plasmid (Table 3). In bacteria of the genus* Vibrio*, the existence of penicillin-resistant encoding plasmid was suggested by Reid and Amyes [33], who described plasmid SAR-1 as capable of hydrolyzing the antibiotics carbenicillin and penicillin G. According to the authors, the most common resistance mechanism to *β*-lactam antibiotics is the production of *β*-lactamase enzymes, which hydrolyze the antibiotic and inactivate it.

We detected two strains with the plasmid resistance to ampicillin (Table 3). Teo et al. [34] associated the ampicillin resistance in vibrios with a possible via of mediation by *β*-lactamase* bla*VHW-1 and* bla*VHH-1 genes of approximately 60 kb present in plasmids.

Molina-Aja et al. [12], in a study of plasmid pattern and antibiotic resistance in* Vibrio* strains isolated from penaeid, detected an incidence of isolates resistant to cephalothin higher than the present study, with 36.1% of the strains carrying resistance to this *β*-lactam. The same authors attributed to a 21,226 pb plasmid the ability to encode the resistance to cephalothin, thus characterizing that resistance as plasmid. This result should not be compared to the ones in the present study, since the resistance to cephalothin observed in 12 strains was, possibly, of chromosome nature.

## 4. Conclusions

The findings of this study support the conclusion that the cultured shrimps can be vehicles of vibrios resistant to *β*-lactam and tetracycline. Thus, the emergence of resistant bacteria to antibacterial drugs may be indicative of the indiscriminate use of these drugs in the cultivation of aquatic organisms. Furthermore, the detection of resistance mediated by mobile genetic elements, plasmids, serves as alert to the possibility of horizontal transfer of antibiotic resistance genes among bacteria.

## Figures and Tables

**Table 1:** Antimicrobial resistance in vibrios isolated from the hemolymph of *Litopenaeus vannamei *shrimp.

Species	*n*	*n*R (%)	Resistance					
			Pen	Tcy	Cpl	Amp	Atm	Cro
*V. navarrensis *	53	38 (71,7)	32	—	3	2	7	1
*V. brasiliensis *	15	9 (60)	9	—	4	1	—	—
*V. parahaemolyticus *	10	10 (100)	10	9	1	3	—	—
*V. xuii *	8	8 (100)	8	—	4	2	—	—
*V. coralliilyticus *	5	3 (60)	3	—	—	—	—	—
*V. cholerae *	4	4 (100)	4	3	—	2	1	—
*V. neptunius *	2	0 (0)	—	—	—	—	—	—
*V. alginolyticus *	1	1 (100)	1	—	—	—	—	—
*V. diazotrophicus *	1	1 (100)	—	—	—	—	1	—
*V. vulnificus *B3	1	1 (100)	1	1	—	—	—	
Total	**100**	**75**	**68**	**12**	**12**	**10**	**9**	**1**

^∗^ *n*: number of isolates. *n*R: number of resistant isolates. Pen: penicillin G; Tcy: tetracycline; Amp: ampicillin; Cpl: cephalothin; Atm: aztreonam; Cro: ceftriaxone.

**Table 2:** Antimicrobial resistance pattern in vibrios isolated from the hemolymph of *Litopenaeus vannamei *shrimp.

Classification	Profile	*n*	Species (number of resistant isolates)
Monoresistance (*n* = 42)	Pen	38	*V. navarrensis* (28), *V. xuii* (4), *V. coralliilyticus *(3), *V. brasiliensis* (2), and *V. alginolyticus* (1)
	Atm	4	*V. navarrensis* (3) and *V. diazotrophicus* (1)

Cross-resistance to *β*-lactam (*n* = 20)	Pen + Cpl	9	*V. navarrensis* (3), *V. brasiliensis* (3), *V. xuii* (2), and *V. parahaemolyticus* (1)
	Pen + Atm	5	*V. navarrensis* (4) and *V. cholerae* (1)
	Pen + Amp	2	*V. navarrensis* (2)
	Cro + Atm	1	*V. navarrensis* (1)
	Pen + Cpl + Amp	3	*V. xuii* (2) and *V. brasiliensis* (1)

Multiple resistance (*n* = 13)	Pen + Tcy	8	*V. parahaemolyticus* (6), *V. cholerae* (1), and *V. vulnificus* (1)
	Pen + Tcy + Amp	5	*V. parahaemolyticus* (3) and *V. cholerae* (2)

^∗^ *n*: number of resistant isolates; Pen: penicillin G; Tcy: tetracycline; Amp: ampicillin; Cpl: cephalothin; Atm: aztreonam; Cro: ceftriaxone.

**Table 3:** Plasmid mediation of antimicrobial resistance in vibrios isolated from the hemolymph of *Litopenaeus vannamei *shrimp.

Strain code	Species	Resistance profile	Posthealing profile	
			Resistant	Sensitive
#1	*V. coralliilyticus *	Pen	—	Pen
#2	*V. navarrensis *	Pen	—	Pen
#3	*V. navarrensis *	Pen + Amp	—	Pen + Amp
#4	*V. navarrensis *	Pen + Amp	Pen	Amp
#9	*V. navarrensis *	Pen	—	Pen
#10	*V. navarrensis *	Pen	—	Pen
#17	*V. navarrensis *	Pen + Atm	—	Pen + Atm
#18	*V. navarrensis *	Pen + Atm	Atm	Pen
#36	*V. navarrensis *	Pen	—	Pen
#56	*V. navarrensis *	Pen	—	Pen
#69	*V. navarrensis *	Pen + Atm	Atm	Pen
#74	*V. brasiliensis *	Pen	—	Pen
#94	*V. brasiliensis *	Pen	—	Pen
#98	*V. brasiliensis *	Pen	—	Pen

^∗^Pen: penicillin; Amp: ampicillin; Atm: aztreonam.
